# Eye Gaze and Aging: Selective and Combined Effects of Working Memory and Inhibitory Control

**DOI:** 10.3389/fnhum.2017.00563

**Published:** 2017-11-27

**Authors:** Trevor J. Crawford, Eleanor S. Smith, Donna M. Berry

**Affiliations:** ^1^Department of Psychology, Centre for Ageing Research, Lancaster University, Lancaster, United Kingdom; ^2^Department of Psychology, Lancaster University, Lancaster, United Kingdom; ^3^School of Psychology, Keele University, Staffordshire, United Kingdom

**Keywords:** aging and cognitive function, antisaccade task, prosaccades, working memory, inhibitory control, attention, dementia, eye-tracking

## Abstract

Eye-tracking is increasingly studied as a cognitive and biological marker for the early signs of neuropsychological and psychiatric disorders. However, in order to make further progress, a more comprehensive understanding of the age-related effects on eye-tracking is essential. The antisaccade task requires participants to make saccadic eye movements away from a prepotent stimulus. Speculation on the cause of the observed age-related differences in the antisaccade task largely centers around two sources of cognitive dysfunction: inhibitory control (IC) and working memory (WM). The IC account views cognitive slowing and task errors as a direct result of the decline of inhibitory cognitive mechanisms. An alternative theory considers that a deterioration of WM is the cause of these age-related effects on behavior. The current study assessed IC and WM processes underpinning saccadic eye movements in young and older participants. This was achieved with three experimental conditions that systematically varied the extent to which WM and IC were taxed in the antisaccade task: a memory-guided task was used to explore the effect of increasing the WM load; a Go/No-Go task was used to explore the effect of increasing the inhibitory load; a ‘standard’ antisaccade task retained the standard WM and inhibitory loads. Saccadic eye movements were also examined in a control condition: the standard prosaccade task where the load of WM and IC were minimal or absent. Saccade latencies, error rates and the spatial accuracy of saccades of older participants were compared to the same measures in healthy young controls across the conditions. The results revealed that aging is associated with changes in both IC and WM. Increasing the inhibitory load was associated with increased reaction times in the older group, while the increased WM load and the inhibitory load contributed to an increase in the antisaccade errors. These results reveal that aging is associated with changes in both IC and WM.

## Introduction

As people get older, they tend to perform many activities more slowly and less accurately. We walk to the shops more slowly; we become more forgetful; we do not hit a golf ball as far in our 60s as we did in our 20s. Executive function is responsible for high-level cognitive operations, including planning, task management, working memory (WM), and inhibitory control (IC). A large body of behavioral research has focused on the effect of natural aging on executive function and has observed age-related deteriorations across a range of cognitive domains including WM and IC (e.g., Anderson et al., 2012; Heilbronner and Münte, 2013). It has been claimed that the natural aging process adversely affects saccadic eye movement functioning (Dowiasch et al., 2015), with the voluntary control over saccadic eye movements showing less resilience to aging (Peltsch et al., 2011). The natural process of aging, however, does not inevitably lead to cognitive decline, and the extent of this cognitive decline varies across tasks and individuals (Friedman et al., 2008; Park and Reuter-Lorenz, 2009; Crawford et al., 2013). Numerous studies have described the effects of aging on saccadic eye movement performance, but with clear inconsistencies; saccade parameters such as reaction times and error rates have been correlated with aging (Abel and Douglas, 2007; Peltsch et al., 2011), whereas, others have shown no difference between older and younger participants (Eenshuistra et al., 2004; Pratt et al., 2006). Clearly, further research is required in order to fully understand the neurocognitive changes involved in human aging. There have been several attempts to locate the decline of specific cognitive operations in aging. However, previous research has often relied on complex tasks with multiple interacting cognitive elements (Rabbit, 2005); therefore in this study we have turned to the well characterized antisaccade task (AST). The current experiment is an attempt to clarify whether, during the aging process, IC, WM, or both processes are associated with the decline in AST performance, which has been previously observed across the literature.

### Aging and Antisaccade Task Performance

Cognitive inhibition can be operationalized as the rejection of a prepotent or highly practiced response, in favor of a more contextually appropriate or desirable response (Butler and Zacks, 2006). The AST presents participants with a central fixation stimulus on a screen, which is then followed (after a brief temporal gap in the commonly employed AST gap task) by a suddenly appearing visual peripheral stimulus. The correct task response requires participants to avoid the instinctive compulsion to look towards the target, and instead to execute a saccadic eye movement to a position on the screen, equidistant from the center of the screen, but in the opposite horizontal direction to the target (an *antisaccade*). The AST is thought to comprise several critical components: remembering the rule of the task; inhibiting the prepotent response to look towards the target; translating the spatial location of the target across to its mirror image location; and executing the oculomotor saccade to what is essentially a blank, undefined location. The composite processes necessary for successful AST responses can be fractionated into sub processes reliant predominantly upon WM or IC (Crawford et al., 2011). While these two cognitive mechanisms may be interrelated, the active suppression of the prepotent saccade to the target, and the active fixation upon the spatial location of the end point of the AST (while self-prohibiting further eye or head movements) rely primarily upon the inhibition system. However, the implementation of the rules of the AST, the translation of the spatial location of the target, and subsequent execution of the antisaccade movement rely more heavily upon WM processes.

In young controls, the AST elicits longer latencies and a higher incidence of directional errors than the prosaccade task (PST). In older adults, these differences are inflated (e.g., Eenshuistra et al., 2004; Crawford et al., 2005; Butler and Zacks, 2006; Peltsch et al., 2011). Despite the prevalence of AST impairments in older adults, the source of these impairments remains unclear. An increased AST latency and the higher error rate found in older adults have been interpreted as a typical age-related decline of inhibitory function (Klein et al., 2000; Sweeney et al., 2001; Peltsch et al., 2011). In agreement with research on the decline of IC with age (Raemaekers et al., 2006; Peltsch et al., 2011), Alichniewicz et al. (2013) demonstrated the reduced ability of older adults to voluntarily inhibits saccadic responses compared to young adults. Additionally, the onset latencies of both prosaccades and antisaccades were significantly longer in healthy older adults, compared to their young counterparts. WM capacity is also suggested to account for the difference between younger and older adults in the AST (Eenshuistra et al., 2004; Crawford et al., 2013). For example, the observed age-related AST impairments could be influenced by declining WM capacity (Baltes et al., 1999), declining levels of activation of task goals, and/or rates of refreshment of crucial task information in WM (e.g., Duncan et al., 1996; Nieuwenhuis et al., 2004), or declining inhibitory efficiency in controlling the amount of task-irrelevant information entering, remaining in, and being filtered out of, WM (e.g., Hasher and Zacks, 1988; Hasher et al., 1999). The accounts differ in the extent to WM (or IC) decline is considered to be a direct source of AST impairments, rather than an indirect, emergent property of cognitive impairment.

Roberts et al. (1994) and Mitchell et al. (2002) presented healthy young adults with a WM task concurrent with an AST, and reported that prolonged latencies and higher directional error rates were observed for young participants that were comparable to that found among much older adults in other studies (Olincy et al., 1997; Sweeney et al., 2001; Eenshuistra et al., 2004). This was interpreted as evidence suggesting that a reduction in WM capacity is the likely source of poor AST performance. In contrast, it has been argued that the failure to inhibit the prepotent response may account for the poorer performance across the older samples in the AST (Butler and Zacks, 2006; Ryan et al., 2006). This is because it is essential to inhibit this response towards a visual stimulus (e.g., Godijn and Kramer, 2006). Butler and Zacks (2006) investigated the extent to which IC could explain age-related AST impairments. Antisaccades that are triggered by the onset of a peripheral stimulus require IC to suppress the prepotent prosaccade response to that stimulus (Theeuwes et al., 1999), while antisaccades signaled by a central directional arrow require endogenous planning, but clearly have reduced inhibitory requirement, as there is no peripheral stimulus to capture visual attention. Thus, a marked increase in AST errors or latencies with age, in a peripheral onset condition, in comparison with a central cue, would suggest support for a specific IC deficit with age. In the Butler and Zacks (2006) study, the older adults generated longer reaction times when the inhibitory load of the AST was increased using the onset of the peripheral stimulus, which is compatible with the hypothesis of an age-related decline in IC. However, the peripheral vs. central cue manipulation yielded no group effect on AST errors between the young and older participants.

#### The Current Study

**FIGURE 1:**
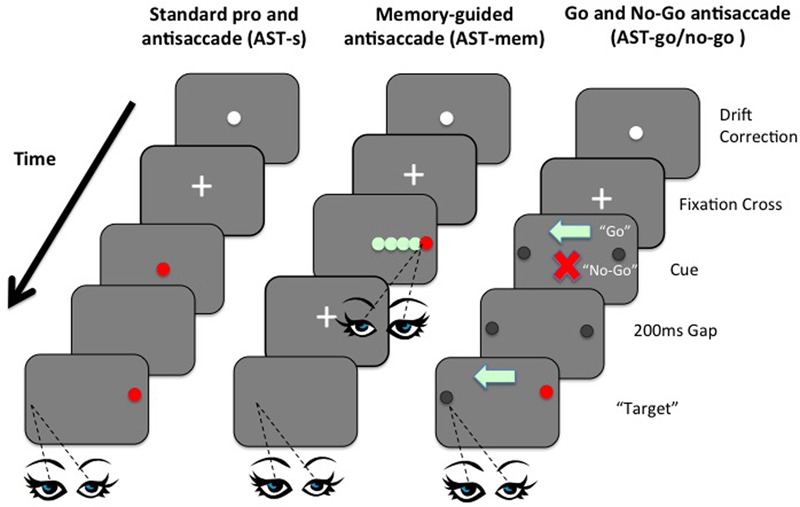
Left panel: Example sequence of screens in the standard prosaccade task (PST) and standard antisaccade task (AST-s). Although visually identical, the difference between the PST and the AST, in terms of the requisite eye movement response towards or away from the target, was made clear in the instructions given to participants before each task. Middle panel: Example sequence of screens in the memory-guided AST task (AST-mem). Right panel: Example sequence of screens in the AST-go/no-go task shows the orders in which screen were presented to participants in the AST-go/no-go task.

Currently, we have various findings, with no consensus. However, no study so far has manipulated WM and IC within the same task domain. Here we present, for the first time, a set of novel ASTs that attempt to manipulate the IC and WM processes independently within the AST. This was achieved by two simple manipulations: First, using a *memory-guided* condition, the memory-load was increased, while removing the usual inhibition of a saccade towards the prepotent target. Memory load was increased by introducing five “to-be-remembered” possible target locations that were embedded within an array of distracters; critically the inhibitory element was simultaneously minimized by the requirement to fixate the target immediately at presentation, before then returning back to the central fixation point ready for the subsequent AST away from the remembered target. By allowing the observer to fixate the target at the time of presentation, the typical requirement to inhibit the saccade to the prepotent target was removed (**Figure 1**). In a second condition (*go*/*no-go*), inhibition was increased while simultaneously minimizing the memory load. In the go/no-go task, participants were presented with an initial fixation stimulus, followed by either a ‘go’ cue (green light) or a ‘no-go’ cue (red cross), before the onset of a target stimulus. On trials where a ‘go’ cue is presented, participants must make a saccadic eye movement to the target (prosaccade) or away from the target (antisaccade). In trials where a ‘no-go’ cue is presented, participants must maintain fixation on the fixation stimulus (i.e., must refrain from making a saccadic eye movement towards or away from the target). Go/no-go paradigms are known to increase inhibition errors, even beyond the standard AST (see Crawford et al., 2005). Critically, the WM load was minimized by presenting a clear visual arrow marker that pointed towards to the correct direction on the “Go” trials, thus eliminating the need to keep this location in mind (**Figure 1**). We employed variants of the AST to selectively load IC or WM, to compare performance on these tasks with performance on the standard AST, across two age groups. Performance was assessed in terms of latencies of correct saccades, spatial accuracy, and the proportion of directionally erroneous saccades on the ASTs (i.e., saccades towards the target, rather than away from the target). The aim was to clarify the extent to which the previously documented age-related impairments on the AST are affected by spatial WM deficits, in contrast to the inhibition of a prepotent response. The AST-go/no-go was designed to assess the effect of an inhibitory load (specifically the ability to successfully inhibit the “urge to go” in the no-go condition), while the memory-guided AST conditions assessed the effect of an increased load on WM. A standard version of the AST (AST-s) acted as a baseline assessment of saccadic performance. The gap paradigm (i.e., where the fixation point is removed 200 ms before the target is presented) was employed in all tasks so as to reduce the possibility of floor effects, whereby the tasks are insufficiently taxing as to elicit any errors from the participants. Including such a temporal gap in eye movement studies increases the distraction error probability in AST trials (Saslow, 1967; Fischer and Weber, 1992; Dorris and Munoz, 1995; Eenshuistra et al., 2004). Kimberg and Farah (2000) proposed in their model that IC was simply an expression or by-product of WM; in other words IC is not a distinct cognitive operation. However, using a single case study methodology, Crawford and Higham (2016) recently discovered evidence for independence and modularity. In this study, we assess IC and WM as part of the cognitive operations underpinning antisaccades in both young and older participants. Saccade latencies and error rates of saccades of older participants were compared to the same measures in healthy young controls.

## Materials and Methods

### Participants

A total of 16 *younger* adults (age range = 18–30) and 15 *older* adults (age range = 50–77) each participated in four conditions. The young group were recruited in person through their attendance at an undergraduate- and masters-level Psychology course at Lancaster University and were reimbursed at a remuneration rate of £6 per hour (with most sessions lasting approximately 90 min). The remaining younger participants were undergraduate-level Psychology students, recruited via an online system and reimbursed in course credits. Approximately half of the older participants were recruited from the Lancaster University Veteran’s Society via advertisements and consequently had all previously studied and/or worked at Lancaster University. The remaining older participants were recruited in person through their attendance at local interest group meetings. All participants were free from psychiatric disorder, as determined by self-report and were free from psychoactive medication. All older participants lived independently in their own homes, and were classified as having no early signs of dementia, or general cognitive impairments according to the Mini Mental State Examination (Molloy et al., 1991). Participants were screened for color blindness using the Ishihara Test (Ishihara, 1973), and for normal or corrected to normal visual acuity using a standardized Snellen chart. The study received ethical approval from Lancaster University ethics committee.

### Cognitive Assessment

Prior to the eye-tracking phase of the experiment, all participants were assessed using a cognitive battery comprising a standardized digit span from the Wechsler Adult Intelligence Scale III (WAIS III; Wechsler, 1997a); Corsi blocks of the spatial span (Wechsler Memory Scale III; Wechsler, 1997b); and the National Adult Reading Test (Nelson, 1982). The older group of participants was additionally screened using the Mini Mental State Examination (Molloy et al., 1991), a brief screening instrument for dementia in order that any participants exhibiting signs of dementia or mild cognitive impairment could be excluded from the experiment.

### Stimulus and Apparatus

Stimuli were displayed against a white background on a 19-inch computer monitor, controlled by a Dell PC, which also recorded the experimental data. Participants were seated at a distance of 57 cm from the screen, with their chins placed on a specially adapted, adjustable chin rest to reduce head movements. Testing was conducted in a quiet, darkened room, to which participants were acclimatized before testing commenced. Eye movements were recorded using an EyeLink II high-speed camera eye tracking system (500 Hz, < 0.5° accuracy) running on a Dell PC. A hand-held computer controller was also connected to the host computer. This permitted participants to control the pace of the presentation of the stimuli for each trial. Participants were required to wear a non-invasive headband supporting three small cameras throughout the experiment. Two cameras recorded eye movements using infra-red technology whereby differential absorbencies of the infra-red energy by the pupil and the sclera permitted the tracking of micro-movements of the pupil. The third tracking camera maintained optimal alignment by monitoring the positioning of four infra-red markers on the computer screen.

### Design

The presentation order of the resulting three conditions was controlled so as to balance any potential effects resulting from asymmetric transfer of experience from one task to the next. Since a PST (where the desired response was for participants to orient their eyes towards the visual target) by definition required less deliberation than the AST (in which participants were required to avert their eyes from the visual target), a PST was always presented before the three ASTs to prevent the additional cognitive processing inherent within the AST impacting on the PST. The two standard tasks (standard PST and AST-s) each comprised 60 trials, split into three blocks of 20, with the location of the visual target (4° to the left or to the right of the central fixation cross) randomized across each block, with the constraint that no more than five consecutive runs of each target location was to be deployed, in order to prevent participants anticipating the target location, or displaying practice effects to that particular target location. The memory-guided task comprised 48 trials, organized into two blocks of 24 trials. The location of the target (1, 3, or 5° to the left or to the right of the central fixation cross) was randomized, with the constraint that no more than five consecutive runs of each target location would be deployed. The go/no-go task comprised 48 trials organized into two blocks of 24. In these conditions, both the target location (4° to the left or to the right of the central fixation cross) and the type of central cue presented (a red cross denoting a no-go trial, or a green arrow denoting a go trial) were randomized, subject to a maximum of five consecutive runs of any one target location and central cue combination. In every condition, participants were offered a brief period of respite after each block of trials, if desired, in addition to a break after each of the three experimental conditions. Performance was assessed in terms of saccade latencies (mean response times after presentation of the target stimulus), spatial accuracy (mean deviation of final eye position from the centre of the intended target location), and error rates (the proportion of trials in which participants moved their eyes in a direction contrary to the task requirements).

### Procedure

A nine-point calibration and validation was conducted prior to each condition to obtain a high degree of tracking accuracy. Participants were presented with standardized on-screen instructions before commencing each condition, and after each block of trials within each condition. Additional verbal clarification was provided where necessary, and participants were permitted to ask for help should they require further explanation. The appearance of a centrally located stimulus consisting of two concentric monochrome circles signaled the start of the trial, and pending initiation from the participants in the form of a button press on the console controller, a central black fixation cross was presented for 200 ms, followed by the experimental stimuli. For each condition, 12 initial practice trials were conducted, and participants were permitted to proceed to the experimental trials subject to satisfactory completion of these practice trials. All tasks incorporated a 200 ms gap between the extinguishing of the central fixation stimulus and the onset of the target stimulus in order to increase the potential for erroneous trials, since these trials were to form part of the basis of the analysis.

## Eye Movement Conditions

### Standard PST Condition

In a standard PST trial, after the task instructions had been presented on screen, the initial central fixation cross was superseded by the target stimulus (a 0.7° diameter, red filled circle) that remained on the screen for 2 s, before disappearing. A 200 ms gap then elapsed, while the screen was completely blank, before the target stimulus reappeared at a location of 4° to the left or right of the screen center, where it remained for 2 s. The 4° target eccentricity has been used in previous saccadic eye movement studies (e.g., Nieuwenhuis et al., 2004) and thus allows for comparisons with the present research. Similarly, the standard timings utilized in the gap paradigm are of 200 ms (*cf*. Crawford et al., 2002; Eenshuistra et al., 2004; Evdokimidis et al., 2006). After a further 700 ms, the target stimulus was extinguished, and the next trial commenced with the appearance of the two concentric circles for drift correction. After 30 consecutive trials, the instruction screen reappeared to remind participants of the requirements of the task at hand and also to allow for short recess in testing should participants so wish. The presentation order of the composite screens in the standard PST (and standard AST-s) condition is shown in **Figure 1** (left panel).

### Antisaccade – Standard Condition (AST-s)

The AST-s condition was visually identical to the PST condition, with the exception of the instructions given to participants. The initial instruction screen requested that participants look to a point in space that they considered being the horizontal mirror-image location of the target stimulus. Further verbal clarification was given to ensure that participants knew that they were required to look to a point equally distant from the center, but in the opposite direction to, the target stimulus. The presentation order of the composite screens in the AST-s condition (and standard PST condition) is represented in **Figure 1** (left panel).

### Antisaccade – Memory-Guided Condition (AST-mem)

The memory-guided condition consisted of two parts: participants were initially presented with an instruction screen then a central fixation cross, which was accompanied after 700 ms by a red, circular target stimulus identical to that used in the standard PST and AST-s conditions, to the left or right. However, this target was adjacent to four distracter stimuli, spaced 1° apart, occupying locations from 1 to 5° of visual angle from the central fixation point. Hence, participants were required to distinguish the target from the distracters and subsequently remember the target location using spatial WM. The distracter stimuli were identical in size and shape to the target stimulus (that is, filled circles of 0.7° diameter), but filled green instead of red. The location of the red target was randomized across possible locations of 1, 3, or 5° from the screen center (i.e., distracters always occupied the locations 2 and 4° from the center). The remaining viable stimulus locations were occupied by the four green distracters, which remained on the screen for 1 s.

The first component of the AST-mem required participants to look directly at the red target stimulus as quickly and accurately as possible, ignoring the green distracters, and then back to the center. In this way, the AST-mem condition eliminated the need for participants to actively inhibit the target, as required in the standard AST-s and in the AST-go/no-go task. In the second part of the task, however, the voluntary saccade was to be executed to the mirror-image location of the previous target location within a blank screen. This variant, the AST-mem condition, thus taxed spatial WM while minimizing the extent to which IC was to be exercised. To reduce the likelihood of older participants neglecting to respond at all, after the delay period (as might be expected according to advocates of the goal neglect hypothesis of aging, e.g., Duncan et al., 1996), a double cross-modality cue was presented to prompt participants to initiate the saccade: the visual offset of the central fixation stimulus, was presented simultaneously with an auditory beep. At the time of the subsequent voluntary saccade, the screen was completely blank, thus creating a situation likely to load more heavily onto WM, since no markers were present to aid the recollection of the previous target location, and the eye movement was programmed according to the spatial representation of the target location in memory. The presentation order of the composite screens in the AST memory-guided (AST-mem) condition is represented in **Figure 1** (middle panel).

### Antisaccade – Go/No-Go Condition (AST – go/no-go)

In this condition, the target was a black filled circle of 0.7° diameter, which appeared at 4° to the left or right of the screen center. The two possible locations of the target were highlighted at all times throughout the trial with faint circular markers, 0.7° in diameter, to reduce the need for participants to maintain location-based representations of the target in WM. Upon extinction of the central cross after 200 ms, one of two stimuli appeared in the same central location: either a red cross or a green arrow, matched in size (approximately 0.7° diameter), and approximate pixel density. A 200 ms gap then elapsed, followed by the appearance of the stimulus to either the left or right as displayed in **Figure 1** (right panel).

Participants were instructed that a central red cross denoted a no-go trial, and consequently participants should remain fixated on this stimulus, making no voluntary eye movements. Participants were instructed that they were required to saccade in the direction indicated by the central green arrow, which would always point in the opposite direction to the location of the target stimulus. The central cue (green arrow or red cross) and the black target remained simultaneously visible for 1 s, before they were extinguished and the trial ended. In this way, the central arrow cues further reduced the WM load, by aiding the orienting of attention, and subsequently, eye movements.

The use of the color red to denote *stop* and the color green to denote *go* was considered to be a pairing that was ecologically valid; participants are likely to have experienced this pairing on many occasions previously (for example at traffic lights or pedestrian crossings). Furthermore, the arrow was considered to be easily comprehensible encouragement to look in the direction indicated by arrowhead, and the cross, an obvious reminder that no eye movements were permitted. Previous research has demonstrated that arrow cues rapidly direct attention towards the direction in which the arrowhead is pointing (Hommel et al., 2001; Tipples, 2002; Butler and Zacks, 2006). Thus, the arrowhead cue pointing towards the opposite visual hemifield to the target location should reduce WM load relative to the standard AST condition, and greatly reduce WM load relative to the memory-guided AST condition. Importantly, the AST-go/no-go still posed an inhibitory load, since participants were required to inhibit the reflexively programmed prosaccade to the sudden-onset of the red distractor. The presentation order of screens in the AST-go/no-go condition is displayed in **Figure 1** (right panel). The mean latencies and mean accuracy deviations of correct saccades, and mean proportion of directional errors were collated for each condition, per participant. Saccades were identified using algorithms applied by DataViewer (SR Research Ltd.,) and then visually inspected by the experimenter to ensure that all saccades were executed at least 80 ms after the appearance of the target stimuli in each condition, and exceeding 1° in amplitude. Latencies of less than 80 ms were deemed anticipatory errors, and such trials were excluded from the analysis. Latencies greater than 800 ms were discounted from the analysis on the grounds that they represented a lapse in concentration and not representative of saccadic performance in general. Visual inspection of the data files enabled trials in which the eye sample visibility was lost by the eye tracker (for example, trials in which the participant may have moved their head, or closed their eyes) to be identified and omitted from data analysis.

The amplitudes of correctly executed saccades were collated, in order that the spatial accuracy of saccades between the conditions, as well as between the two age groups could be assessed. Accuracy was calculated as the absolute error of the eye landing position in relation to the target. Trials in which participants looked in a direction contrary to the task requirements (i.e., where participants look towards the target in AST conditions; where participants looked towards the target in no-go trials of the AST-go/no-go conditions; or where participants failed to look at the target in PST) were classed as errors, and were analyzed separately to valid trials. The number of such directional errors was recorded, and the proportional error rate was calculated as the absolute number of errors divided by the number of valid trials recorded for that participant in that condition. Repeated-measures analyses of variance using SPSS (v22) were conducted in order to distinguish the effects of WM and IC on the different tasks. Mauchly’s test was used to examine any violations of sphericity in the repeated-measures analyses. Where Mauchly’s failed to reject the null hypothesis of sphericity, the Greenhouse-Geisser, Huyn-Feldt, and Lower-Bound corrections were examined using the corrected degrees of freedom. As all the effects remained statistically significant (*p* < 0.01), for both the sphericity assumed and sphericity corrected, we only report the *F*- and *p*-values for the sphericity unassumed analyses. The Levene’s test was used to examine the assumption of homogeneity of variance. Where this assumption was not satisfied, we re-examined the analyses using square-root transformation. As the pattern of results was unchanged and remained significant at *p* < 0.01, we report the statistics for the untransformed data.

## Results

### PST

First, we conducted a one-way ANOVA on the PST data to determine whether the groups were comparable in the baseline, control task, where there was no IC or WM load. The mean latencies and spatial accuracy for the PST baseline task were comparable in the older group (mean latency = 171 ms, *SD* = 43; mean spatial accuracy = 0.66°, *SD* = 0.27) and younger group (mean latency = 154 ms, *SD* = 25.1; mean spatial accuracy = 0.57°, *SD* = 0.14). The ANOVA revealed that there was no effect of age group on prosaccade latencies [*F*(1,30) = 1.76, *p* = 0.195] or spatial accuracy [*F*(1,30) = 1.324, *p* = 0.259]. This confirms that there was no general decline in motor processing speed or spatial accuracy with age.

### AST/PST Saccade Latency

**FIGURE 2:**
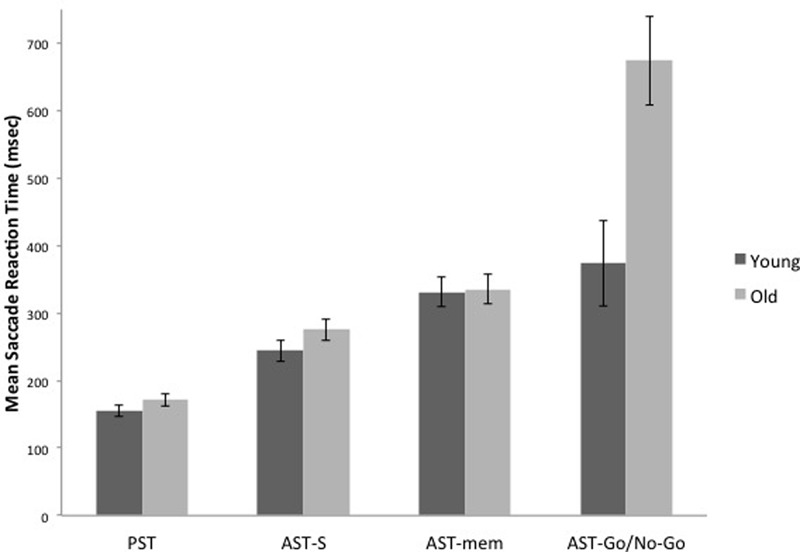
It shows the means of saccade latencies for the younger and older participants in the prosaccade (PST), the standard antisaccade (AST-s), memory-guided antisaccade (AST-mem), and go/no-go antisaccade tasks (AST-go/no-go). Older participants have substantially longer latencies in the AST-go/no-go in comparison with the younger participants. Error bars represent standard errors.

The WM hypothesis predicts an interaction between age group and the tasks; according to this hypothesis, the older group should reveal disproportionate slowing on the AST-mem task, due to the fact that the AST-mem places a high load on spatial memory (together with a low inhibition load ***–*** eye-movements were permitted to the prepotent target). The IC hypothesis also predicts an interaction between age group and task. However, according to the IC hypothesis, the older group should show a greater slowing in the AST-go/no-go, due to the fact that this task demands a higher level of IC than the AST-s (together with a low load on WM). Therefore, we submitted the latency data to a 2 (age group) ***×*** 4 (task) mixed ANOVA. This analysis revealed that there was an overall increase in mean reaction times for the older adults [Group effect: *F*(1,29) = 12.716, *p* = 0.001, η^2^ = 0.305]. Critically, there was a significant task ***×*** group interaction [*F*(3,87) = 8.008, *p* = 0.001, η^2^ = 0.216]. In support of the IC hypothesis, **Figures 2** and **4** reveal that the saccade latencies of the older groups were disproportionately slowed in the AST-go/no-go task. To determine the source of this interaction, we conducted a series of t-tests to evaluate the effect of age ***group*** in each of the ***three*** AST tasks. The *t*-tests revealed that there was a significant effect of age group in the AST-go/no-go task [*t*(29) = ***-***3.282, *p* = 0.003], but there was no age ***group*** effect for the AST-s [*t*(29) = ***-***1.419, *p* = 0.167], or AST-mem tasks [*t*(29) = ***-***0.128, *p* = 0.899].

### AST Errors

**FIGURE 3:**
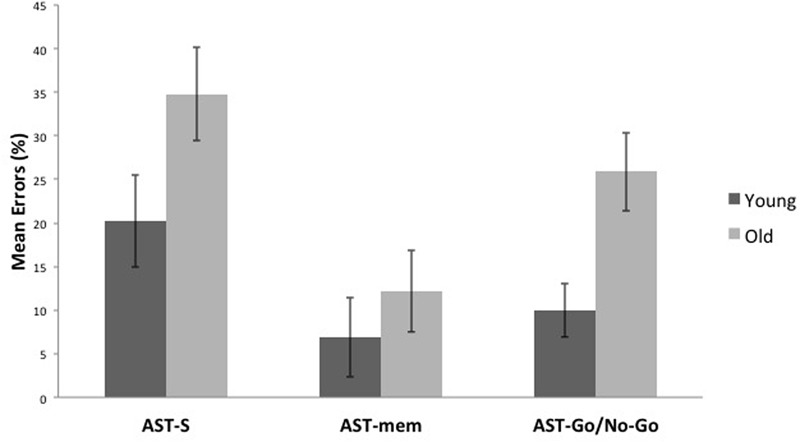
It shows that older participants make a higher proportion of errors than younger participants in all, the standard antisaccade (AST-s), memory-guided antisaccade (AST-mem), and go/no-go antisaccade tasks (AST-go/no-go). Error bars represent standard errors.

**FIGURE 4:**
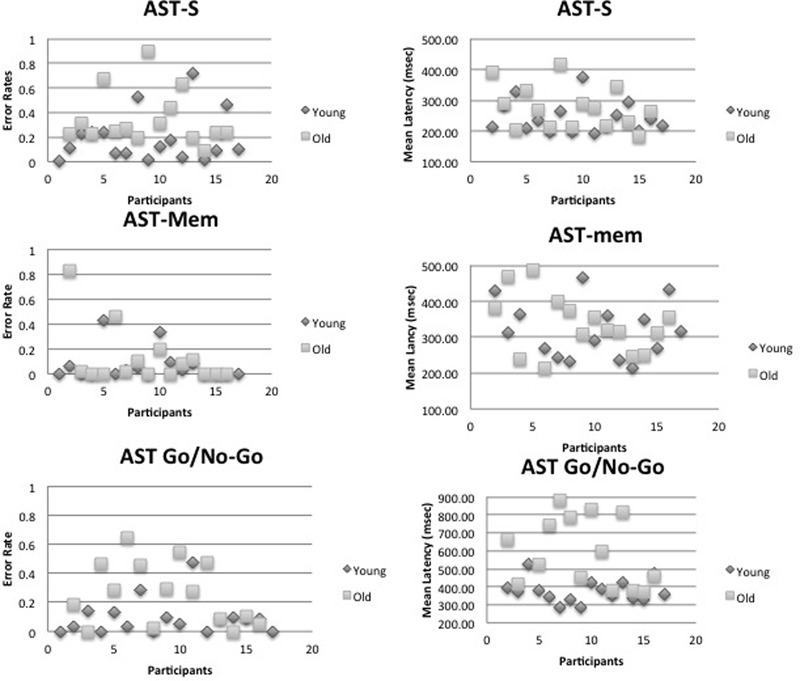
It shows that individual mean errors and latency scatter plots for the young and old age group. Top panel – the standard antisaccade (AST-s), middle panel – memory-guided antisaccade (AST-mem), and bottom panel – go/no-go antisaccade tasks (AST-go/no-go).

Compared to the young group, the older group committed more errors across all the AST conditions [*F*(1,29) = 8.122, *p* = 0.008, η^2^ = 0.219]. Unsurprisingly, there was a significant task effect [*F*(2,58) = 7.391, *p* = 0.001, η^2^ = 0.203], with the AST-mem yielding the smallest proportion of errors (recall that participants were permitted to look immediately towards the prepotent target at its target onset). The AST-s yielded the most errors, presumably because this task did not facilitate either memory (*cf.* AST-go/no-go) or the release from inhibition (*cf.* AST-mem). This analysis revealed that there was no significant interaction of the task with age group (**Figures 3, 4**).

### AST Accuracy

There was no effect of age group on the accuracy of saccades across the three ASTs [*F*(1,29) = 0.901, *p* = 0.258, η^2^ = 0.044]. Older groups show no deterioration in accuracy in any of the three variants of the AST (AST-s: Old = 1.49°, *SD* = 0.93; Young = 1.66°, *SD* = 0.82; AST-mem: Old = 0.66°, *SD* = 0.13, Young = 0.56°, *SD* = 0.25; AST-go/no-go: Old = 1.17°, *SD* = 1.18, Young = 0.71°, *SD* = 0.32). There was a significant main effect of AST task, in a similar fashion to the errors data reported above: the least spatially accurate saccades were generated in AST-s, and the highest spatial accuracy was found in the AST-mem [*F*(3,87) = 15.246, *p* = 0.000, η^2^ = 0.34].

#### Psychometric Cognitive Measures

There were no significant effects of age group on any of the standard psychometric measures. For both age groups combined, there was a significant correlation between digit and verbal span (Pearson’s *r* = 0.707, *p* < 0.01); spatial span was also significantly correlated with NART scores (*r* = 0.463, *p* < 0.01). The saccade latencies in the AST-s (*r* = **-**0.468, *p* < 0.01) and AST-go/no-go (*r* = **-**0.477, *p* < 0.01) were both significantly, negatively correlated with spatial span. There were no significant correlations between AST error rates and the psychometric measures for the young or old groups. However, for the young group, there was an unexpected correlation (*r* = 0.69, *p* = 0.003) between error rates in the AST-go/no-go task and the NART. This suggests that this demanding inhibition task yields fewer errors in those young people with greatest amount of cognitive reserve, and yields more errors in those with lower cognitive reserve as reflected in the NART premorbid IQ measure.

## Discussion

Theories about the cause of the age-related differences on the AST have largely centered around two sources of cognitive dysfunction: IC and WM. According to Hasher et al. (1999), Hasher and Zacks (1988), and Crawford et al. (2011) a reduction in IC with advancing age could affect performance on cognitive tasks in various ways: an age-related deterioration of an inhibitory filter, acting as a gateway into WM, could lead to a decline in the efficiency in which task-irrelevant information is prevented from entering WM; a reduction in the ability to suppress irrelevant information that has entered WM could prevent distracting information from being inhibited, and thus increase the interference effects of this irrelevant information on the current cognitive task. Therefore, the IC hypothesis claims that the age-related impairments in tasks involving WM are causally related to *inhibitory* cognitive mechanisms deteriorating with age, and not the other way round. The alternative WM-led approaches claim that this should be reversed; it is primarily a deterioration of WM that is responsible for age-related AST impairments.

Before turning to the aging effects on AST cognitive control, it is worth noting several features of the prosaccadic eye movements that were preserved in the older population. The PST data allows for a baseline comparison against the three ASTs across the age groups. The reaction times of prosaccades were relatively well preserved in the older group (mean = 175 ms vs. young group mean = 154 ms), showing that processing speed was preserved in the older group. It is important to note that there was no evidence that these fast reaction times were achieved at the expense of accuracy. The older group (mean = 0.64°) was not significantly different to the prosaccade spatial accuracy of the young group (mean = 0.58°). The frequency of prosaccade errors was negligible for both age groups. This confirms that prosaccades are fast, automatic responses that are relatively well preserved in healthy aging. As expected, prosaccade latencies were significantly faster than AST latencies for both the young and older adults, reflecting the additional processing component required for motor programming in the AST (Hallett, 1978).

According to previous research, older people generate increased latencies and/or an increase in the proportion of directional errors in the AST (e.g., Fischer et al., 1997; Eenshuistra et al., 2004). The aim of the present research was to determine whether WM or IC was the source of this effect. The key findings revealed the following: First, the older adults were substantially slower in the AST-go/no-go task. Clearly, the additional inhibitory load had a disproportionate effect on the saccade slowing for the older group. Saccade latencies in AST-go/no-go task were markedly slower than AST-mem latencies. This slowing was probably due to the element of response uncertainty in the AST-go/no-go task. This appears to have resulted in a general response hesitancy presumably due to the 50% chance that any given trial will require an eye movement away from the target, or complete inhibition of the saccade. Participants did not know whether they would be required to execute an antisaccade, or to maintain fixation on the central cross.

Second, the older group generated more errors in each of three ASTs compared to the young group. In contrast to response latencies, this age group effect was of a similar magnitude across the ASTs. The proportion of errors differed between the AST-mem and the AST-go/no-go. As expected, higher error rates were generated in AST-s due to the absence of any cues to facilitate either WM or IC. This implies that both WM and IC contributed to the increased error rates of the older group. In contrast, the spatial accuracy of the saccades was similar in the two groups.

No significant differences were observed in the psychometric measures, between the two age groups. Since some of these measures purportedly reflected WM efficiency (the digit span forward; digit span backward; spatial span forward; and spatial span backward), this suggests these psychometric measures are relatively insensitive to the AST effects on aging. This result has been found previously. Surprisingly in the Eenshuistra et al. (2004) experiment reported earlier, conclusions were drawn in favor of a deterioration of WM as the key source of age-related AST impairments, under the assumption that WM deficits among older participants may only noticeably affect cognitive task performance under conditions of competing task responses (e.g., Roberts et al., 1994). Thus, the WM measures derived from the AST conditions of the various tasks may be more sensitive to subtle age-related cognitive changes in healthy adults.

The primary question this research addressed was whether it was possible to detect independent influences of IC and WM processes on the decline in the age-related executive control of eye gaze. A significant interaction was observed between age group and the AST-go/no-go latencies. The AST-go/no-go demands a greater IC than the standard AST; the substantive effects in the older cohort provide strong evidence that it is the increased inhibitory load (rather than WM) that was problematic for this older group. This finding supports the Hasher and Zacks (1988) *Inhibition Hypothesis*. However, AST errors were increased in all of the ASTs. This suggests that, for the older group, neither the selective facilitation of WM nor IC was sufficient to eliminate AST errors. Apparently, both of these operations contribute to the errors that older people experience in the ASTs. The error data demonstrates that healthy aging is associated with changes in both IC and WM. Reduced IC was related to the slowing in the speed of reaction times in the older groups, while reduced WM and IC both contribute to the increased errors.

## Conclusion

An understanding of the specific mental operations that underlie the changes in aging and dementia is of theoretical and practical importance. Clearly, valid cognitive models of aging require a clearer understanding of the core cognitive processes and their interrelationships (Crawford et al., 2011). This will also help to develop effective and preventative strategies for maintaining cognitive health in old age.

## Data Accessibility

The data created during this research are openly available from Lancaster University data archive at http://dx.doi.org/10.17635/lancaster/researchdata/189.

## Ethics Statement

This study was carried out in accordance with the recommendations of Lancaster University Psychology Ethics Committee with written informed consent from all subjects. All subjects gave written informed consent in accordance with the Declaration of Helsinki and ethical guidelines of the British Psychology Society. The protocol was approved by the Lancaster University Psychology Ethics Committee.

## Author Contributions

TC designed the experiments; DB recruited and tested the controls; TC, ES, and DB analyzed the eye-tracking and psychometric data; TC, ES, and DB edited the manuscript. TC and DB wrote the manuscript.

## Conflict of Interest Statement

The authors declare that the research was conducted in the absence of any commercial or financial relationships that could be construed as a potential conflict of interest. The reviewer DK and handling Editor declared their shared affiliation.
